# Fibrin biopolymer sealant and aquatic exercise association for calcaneal tendon repair

**DOI:** 10.1590/ACB360407

**Published:** 2021-05-21

**Authors:** Silvia Maria Cardoso Magalhães Hidd, Carla Roberta Tim, Eneas de Freitas Dutra, Antônio Luiz Martins Maia, Lívia Assis, Rui Seabra Ferreira, Benedito Barraviera, José Figueiredo Silva, Marcello Magri Amaral

**Affiliations:** 1 MSc. Universidade Brasil – Scientific and Technological Institute – Sao Paulo (SP), Brazil.; 2 PhD. Universidade Brasil – Scientific and Technological Institute – Sao Paulo (SP), Brazil.; 3 PhD. Universidade Estadual do Piauí – Center for Research in Biotechnology and Biodiversity – Teresina (PI), Brazil.; 4 PhD. Universidade Estadual Paulista – Center for the Study of Venoms and Venomous Animals – Botucatu (SP), Brazil.; 5 PhD. Universidade Estadual Paulista – Botucatu Medical School – Botucatu (SP), Brazil.

**Keywords:** Biopolymers, Tendons, Achilles Tendon, Rats

## Abstract

**Purpose:**

The aim of this work was to analyze the effect of fibrin biopolymer sealant (FS) associated or not to aquatic exercise (AE) on the calcaneal tendon repair.

**Methods:**

Forty-four female Wistar rats were randomly divided into four experimental groups: Lesion control (L), Lesion and FS (LS), Lesion and AE (LE) and Lesion and FS associated to AE (LSE). The edema volume (EV), collagen ratio, and histopathological analysis were evaluated after 7, 14, and 21 days of partial tendon transection.

**Results:**

The EV was statistically reduced for all treatment groups after 7 and 21 days when compared to L group. The LS and LSE had the highest EV reduction after 21 days of treatment. The FS group didn’t induce tissue necrosis or infections on the histopathological analysis. It was observed tenocytes proliferation, granulation tissue and collagen formation in the tendon partial transection area in the FS group. The LSE demonstrated higher amount of granulation tissue and increased the collagen deposition at the injury site.

**Conclusions:**

Our data suggests that the therapeutic potential of the association of heterologous fibrin biopolymer sealant with aquatic exercise program should be further explored as it may stimulate the regeneration phase and optimize calcaneal tendon recovery.

## Introduction

Tendons are exposed to extreme mechanical demands of the human body because they are responsible for transmitting muscular forces to the skeleton and allowing body movement^1^. The incidence of tendon rupture had increased in the last four decades, more often in males 30 to 50 years old^2^. The calcaneal tendon is the most commonly affected, with an annual incidence of 40 per 100,000 person-years^3^. Thus, tendon traumas still constitute a big challenge in orthopedic medicine^4^.

The ruptured tendon can be treated with surgical and nonsurgical therapies, although there is no consensus regarding the optimal treatment protocol. Non-operative treatment is associated with a higher risk of future tendon disruption^5^–^7^. Thus, orthopedic surgeons have applied the invasive surgical repair for acute calcaneal tendon rupture^8^,^9^. However, this procedure may result in devastating surgery-specific complications, such as infection or sural nerve injury. Therefore, several strategies have been studied to obtain a minimally invasive approach and to minimize the surgical risks^10^–^12^.

One alternative is to glue the tissues using biopolymers with adhesive and hemostatic properties, prevent fluid loss, facilitate adherence, and eliminate potential future fistulas. The heterologous fibrin biopolymer sealant (FS), derived from snake venom, has hemostatic, adhesive, sealant, scaffold, drug delivery properties, and has become widely used in experimental surgery^13^–^19^. During the polymerization, FS develops a robust three-dimensional fibrin network configuration, acting as a support for cell adhesion and proliferation, stimulating the healing process^13^,^16^,^20^,^21^. This is a non-commercial experimental product, with low-cost, which, as a result, improves efficiency reducing surgery time, and complications^16^–^18^.

Furthermore, early mobilization and functional rehabilitation decrease the re-rupture rate instead of an aggressive surgical procedure. Aquatic exercises (AE) are a conservative option for treating tendon injury. The tendons repair process has a good response to aerobic exercise, such as swimming and running^22^. Tendinous cells respond to physical exercise by producing growth factors, IGF-I and TGF-β1, involved in the synthesis of collagen and other extracellular matrix (ECM) components^23^. AE has a positive effect on reducing muscle pain and spasms, as well as on maintaining physical resistance^24^.

Despite the stimulatory effects of FS and AE training on tissue repair treatment demonstrated by many authors, there is a lack of information about the interaction of these approaches in the tendon healing. In this context, the aim of this work was to analyze the effect of FS, associated or not to AE, on the calcaneal tendon repair.

## Methods

This work was conducted after Ethics Committee on Animals Use approval under protocol number 0326/2019.

Eighty-four 60 days old female Wistar rats (*Rattus norvegicus*), weighing 206 ± 24 g, were analyzed. The animals were placed in plastic cages with sawdust bedding, in the amount of two animals per cage, and were allowed to move freely in the cages with free access to commercial food and water. All animals were submitted to a preconditioning AE. The animals were randomly divided in four experimental groups: lesion control (L), heterologous fibrin biopolymer sealant (LS), aquatic exercise (LE), and heterologous fibrin biopolymer sealant associated to aquatic exercises (LSE).

In the L group, the calcaneal tendon partial transection (CTPT) was induced and did not receive any additional treatment. In the LS group, CTPT was induced and surgically glued with FS. In the LE group, CTPT was induced and the animals were treated with AE during the recovery period. Finally, in the LSE group, CTPT was induced and it was surgically treated with FS and AE during the recovery period.

The animals were evaluated after 7, 14, and 21 days of the surgical CTPT (7 animals per group, per period). All animals were euthanized by an overdose of sodium thiopental (100 mg/kg) by intraperitoneal injection after the evaluation period.

### Heterologous fibrin biopolymer sealant

The FS derived from snake venom used in this study is composed of thrombin-like fraction purified from *Crotalus durissus terrificus* venom, cryoprecipitate of buffalo blood, and calcium chloride (CEVAP, UNESP – Brazil). The use of FS followed the manufacturer’s instructions. In brief, the product was provided in three microtubes: Fraction I – vial composed of serine protease; Fraction II – vial composed of cryoprecipitate containing coagulation factors (factor V, VIII, and von Willebrand), in addition to fibrinogen; Diluent – vial containing a stable solution of calcium chloride. For more details, refer to patent numbers BR1020140114327 and BR1020140114360. The compound was maintained at –20 ºC until application. The compound was thawed, reconstituted, mixed, and applied in each transected tendon, in order to generate a stable clot with a dense fibrin network^25^–^27^.

### Surgical calcaneal tendon lesion induction

The right posterior paw of each animal was trichotomized and asepticized with alcohol 70%. The calcaneal tendon was exposed with a longitudinal incision (3 cm) on the animal’s skin, and a partial transection of the tendon (standardized as half of the tendon cross-section) was performed by a surgical scalpel^4^.

For LS and LSE groups, the calcaneal tendon was glued with 40 ?L of FS^16^. After the application, a polymerized stable clot was formed, constituted by a stable and dense fibrin network^4^. After surgical intervention, all animals were sutured and accommodated in the bioterium.

### Aquatic exercise protocol

The AE was divided into adaptation and post-surgery stages. The first was performed five times per week during 15 days before the CTPT surgical process. All animals were submitted to an increasing AE period (up to 10 min per day on the end of the period) and load (up to 10% of its weight), in order to adapt to the liquid medium. The AEs were performed in an 100 L capacity tank, filled with 40 cm depth water at the temperature of 32 °C.

The post-surgery stage was applied five times per week for 10 min only for LE and LSE groups after CTPT for the treatment period (7, 14, and 21 days, in accordance to its group)^4^. The animals were weighed once a week to establish the lead load (10% of its weight) and have fixed, in the pectoral region, a custom vest during exercise^28^,^29^, avoiding animal flotation^30^.

### Edema volume evaluation

The edema volume (EV) of the animal paw was measured by a custom plethysmometer. A pen-mark 0.5 cm above the incision was used to standardize the paw immersion point in the plethysmometer. The difference between before and after surgical intervention volume was due to the inducted edema. The EV was measured 24 h, and 7, 14, and 21 days after CTPT induction.

### Tissue collection and preparation

The tenotomy process was performed to remove the calcaneal tendon and its muscle insertion for histological analysis. Using an automatic tissue processor (PT05 TS, Lupetec™ – Sao Paulo, Brazil), the tendon was fixed in 10% formalin for 24 h, and then washed in running water for other 24 h. The tendons were dehydrated in a growing solution of ethyl alcohol (70, 90, and 100%). The pieces were diaphanized in an alcohol/xylol solution (1:1), followed by two baths of pure xylol. After processing, the samples were embedded in paraffin.

Four longitudinal histological sections (5 ?m thick) in each block were cut using a rotating microtome (MRP 09, Lupetec™ – Sao Paulo, Brazil). The slides were stained with hematoxylin and eosin (HE) and Masson’s trichrome (MT).

### Histopathological evaluation 

For histopathological analysis, the HE stained slides were imaged with a light microscope (Olympus, Optical Co. Ltd™ – Tokyo, Japan). A pathologist evaluated the lesion site for presence of fibroblasts and tenocytes; inflammatory process; fibrinoid tissue; ECM organization; and granulation tissue.

Two specialists classified the same images using a modified Bonar score^31^ for tendinopathy. Four histological parameters were evaluated: cell morphology; cellularity; vascularization; and fundamental substance accumulation. It was classified in a 4-point scale, where: 0 = normal;1 = slightly abnormal; 2 = abnormal; and 3 = markedly abnormal. The final score is the sum of each parameter analyzed.

### Collagen quantification

Six images of the MT-stained histological slices were obtained by an optical microscope for each studied group. The images were analyzed by a custom software (adapted from Quinn *et al.* 32) implemented in MATLAB R2019b (MathWorks™ – United States) to obtain the collagen quantification. Briefly, the color channels (Red – R;Green – G; and Blue – B) of the image were separated, and B/R and G/R ratios were computed to obtain two masks, using a threshold of 15% of the highest B value. The masks were combined to obtain the collagen ratio in each image.

### Statistical analyses

Statistical analyses were performed using Minitab 18 statistical software. The data normality was evaluated using the Anderson-Darling test. The non-parametric Kruskall-Wallis test was used to compare the groups using 5% as significance level.

## Results

### Edema volume analysis

The EV after 24 h of surgery presented no statistical difference (p-value = 0.023 for the null hypothesis that all means are equal) between groups.

**Figure 1:**
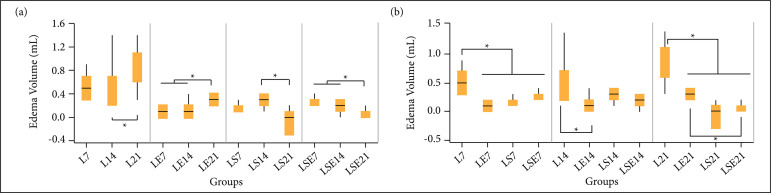
Edema volume of the paw submitted to the surgical induction of partial transection of the calcaneal tendon. (a) Grouped by treatment; and (b) grouped by period. L = control group; LE = aquatic exercise group; LS = fibrin biopolymer sealant group; LSE = fibrin biopolymer sealant and aquatic exercise group.

Fig. 1 presents the EV for each studied group after 7, 14, and 21 days, ordered by its experimental group (Fig. 1a) and by its period of study (Fig. 1b) to facilitate its interpretation.

*p < 0.05.

From intergroup comparison (Fig. 1b), after 7 days, all treated groups (LE7, LS7, and LSE7) presented statistically lower EV (p-value < 0.002) compared to the control group (L7). After 14 days, only LE14 presented a statistically lower EV value (p-value = 0.030) compared to control L14. However, after 21 days, all treated groups (LE21, LS21, and LSE21) presented EV values statistically lower (p-value < 0.001) than control L21. The association of FS and AE (LSE21) showed a significantly lower EV value (p-value = 0.0029) than the group treated only with AE (LE21).

From intragroup comparison (Fig. 1a), the control group (L21) presented statistically higher values than L14 (p-value = 0.04), showing that the lesion without treatment does not evolve satisfactorily. The AE group showed statistical differences between 7 (LE7), and 14 days (LE14), from day 21 (LE21) (p-value = 0.009 and 0.0096, respectively), showing an increase on the EV when this treatment was applied. In the FS groups, a statistical difference was observed between the 14 (LS14) and 21 days (LS21) (p-value = 0.0014) groups, showing a decrease on the EV when this treatment is applied. Finally, in the FS and AE group, a statistical difference was observed between 7 (LSE7), and 14 days (LSE14), from day 21 (LSE21) (p-value = 0.0048), showing a decrease in the EV when this treatment was applied.

### Histological evaluation

**Figure 2:**
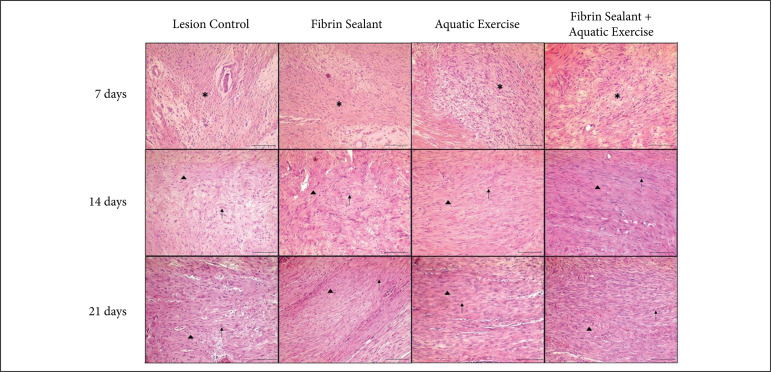
Representative HE histological aspect.

Qualitative histopathological analysis of the calcaneal tendon stained with HE demonstrated that the partial transection was followed by a typical tendon repair process, with the participation of the endotendon (Fig. 2).The proliferation of tenocytes in the area under repair in the endotendon region was similar for all groups. This indicates the chondrocyte differentiation characteristic of the tendon repair process.

* = granulation tissue occupying the region of partial transection; ▲ = loose extracellular matrix; ↑ = tenocytes.

#### 7 days

After 7 days of treatment, all groups showed proliferation of tenocytes between the collagen fibers in the endotendon close to the border of the CTPT, as well as nucleus becoming more ovoid to round in shape without conspicuous cytoplasm. The region resulting from the CTPT was occupied by granulation tissue with variable characteristics between the groups.

In the control group (L), the CTPT region showed loose granulation tissue with tenocytes arranged in disorganized bundles. In the endotendon, located on the margins of the region of CPTP, it was observed an intense proliferation of ovoid tenocytes positioned in rows between the collagen fibers. A small region of the CTPT area was occupied by young granulation tissue, ECM with presence of edema, blood vessels, fibrinoid material, and inflammatory infiltrate composed of neutrophils and macrophages.

In the LE group, the CTPT region was occupied by mature granulation tissue, with rare inflammatory cells, and presented tenocytes arranged in more organized bundles. The presence of intense proliferation of ovoid tenocytes was also observed in the marginal endotendon.

In the LS group, macrophages and fibroblasts were observed at the edges of the CTPT region. Tenocytes organized in rows were present, with sparse cytoplasm and thin nucleus, arranged in bundles parallel to the great axis of the tendon. Numerous ovoid tenocytes have been observed in the marginal endotendon.

Similarly, the LSE group, showed macrophages and tenocytes at the edges of the CTPT region, which was predominantly filled with mature tenocytes arranged in bundles parallel to the great axis of the tendon. There was also an intense proliferation of ovoid tenocytes in the marginal endotendon.

#### 14 days

After 14 days, similar histological findings were observed presenting better tenocytes organization.

The control (L) group showed the CTPT region filled by tenocytes arranged in wavy bundles in a loose ECM. In the LE group, the CTPT region was filled by tenocytes arranged in wavy bundles in a denser ECM compared to controls. Likewise, the LS group showed marked proliferation of tenocytes occupying the region of CTPT, organized in bundles parallel to the large axis of the tendon, and with dense ECM. In the LSE group, it was observed the presence of a denser ECM with intense proliferation of tenocytes arranged in bundles parallel to the large axis of the tendon.

#### 21 days

After 21 days of treatment, in all groups, the CTPT region showed proliferation of ovoid tenocytes more evident in the endotendon, with differences between groups.

The control group presented the region of CTPT filled by intense proliferation of tenocytes, arranged in bundles parallel to the great axis of the tendon and a small dense ECM. In the LE group, the proliferation of tenocytes was especially intense, occupying the region of CTPT and extending to the epitendon. The LS group presented an increased proliferation of tenocytes comparable to the control and LE groups. In the LSE group, the proliferation of tenocytes was more accentuated than in the other groups, with tenocytes arranged in parallel and compact bundles.

None of the groups exhibited a complete reorganization of the tendon structure in 21 days of treatment. However, the histological findings indicate that the use of FS, alone or in combination with AE, has beneficial effects in the treatment of experimental CTPT in rats.

The histological analysis findings were classified in accordance to the Bonar score (Table 1) for each group and studied period.

**Table 1:** Histological Bonar score.

	Cellular morphology	Cellularity	Vascularization	Fundamental substance	Bonar score
L7	2.0	2.0	1.5	1.9	7.0 ± 0.5
LE7	1.2	1.6	0.7	1.6	5.3 ± 0.3*
LS7	1.2	1.8	0.7	2.0	5.8 ± 0.3*
LSE7	1.1	1.6	1.2	2.3	6.4 ± 0.6*
L14	1.4	1.3	2.3	2.1	7.2 ± 0.4
LE14	1.0	1.5	1.6	2.3	6.6 ± 0.3
LS14	0.9	1.3	1.5	2.1	5.8 ± 0.3^a^
LSE14	1.0	1.3	1.3	1.8	5.6 ± 0.5^a^,^b^
L21	1.0	1.3	1.4	1.9	5.7 ± 0.3
LE21	1.2	1.5	1.1	1.3	5.3 ± 0.4
LS21	1.2	1.8	0.8	1.5	5.4 ± 0.5
LSE21	1.1	1.2	0.8	1.4	4.7 ± 0.4^c^

* Statistical difference in comparison to L7;

a Statistical difference in comparison to L14;

b Statistical difference in comparison to LE14;

c Statistical difference in comparison to L21.

**Figure 3:**
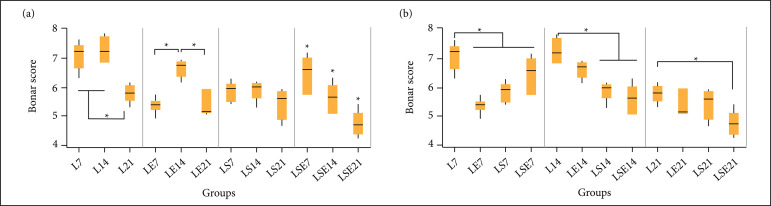
Total Bonar score. (a) Grouped by treatment; and (b) grouped by period. L = control group; LE = aquatic exercise group; LS = fibrin biopolymer sealant group; LSE = fibrin biopolymer sealant and aquatic exercise group.

Figure 3 presents the Bonar score for each studied group, ordered by its experimental group (Fig. 3a) and by its period of study (Fig. 3b) to facilitate its interpretation.

*p < 0.05.

In the intergroup comparison (Fig. 3b), the Bonar scores value were significantly lower in the treated animals after 7 days of CTPT—LE7 (p-value < 0.0001); LS7 (p-value < 0.0001); and LSE7 (p-value < 0.0001)—when compared to control group L7. Likewise, after 14 days, the treated groups, LS14 (p-value < 0.0001) and LSE14 (p-value < 0.0001), had also significantly lower scores compared to the control group L14. Finally, after 21 days, the LSE21 presented a statistical difference (p-value = 0.005) compared to the control group L21.

In the intragroup comparison (Fig. 3a), the control group L21 showed statistical difference (p-value < 0.0001) compared to L7 and L14. In the AE treated group, LE14 showed statistical difference compared to LE7 and LE21 (p-value < 0.0001). The group treated with FS did not show statistical difference between the treatment periods (LS7, LS14, and LS21). The group treated with FS and AE presented a statistical difference between all studied times (LSE7, LSE14, and LSE21), showing a consistent reduction on the Bonar score.

### Collagen quantification

**Figure 4:**
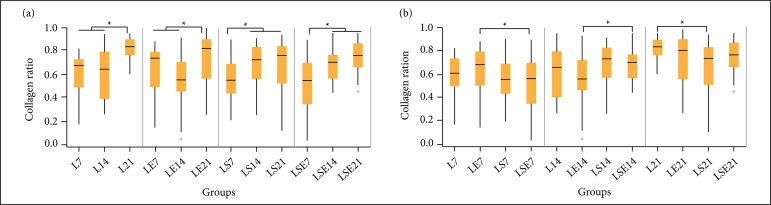
Collagen ratio obtained by the Masson’s trichrome (a) by group and (b) by experimental period. L = control group; LE = aquatic exercise group; LS = fibrin biopolymer sealant group; LSE = fibrin biopolymer sealant and aquatic exercise group.

Fig. 4 presents the collagen ration obtained by the MT stained histological slices for each studied group, ordered by its experimental group (Fig. 4a) and by its period of study (Fig. 4b) to facilitate its interpretation.

*p < 0.05.

For the intragroup comparison (Fig. 4a), the control group presented statistical differences of 7 (L7) and 14 days (L14) from day 21 (L21) (p-value < 0.000001 and 0.00007, respectively). Likewise, in the LE group, the same significant differences were found (p-value = 0.025 comparing LE21 to LE7 and p-value = 0.00011 comparing LE21 to LE14). For the LS group, a statistical difference was observed between LS7 and LS14 (p-value = 0.004), as well as between LS7 and LS21 (p-value = 0.009). Similarly, in the LSE group, a statistical difference was observed between LSE7 and LSE14 (p-value = 0.004) and between LSE7 and LSE21 (p-value = 0.00002).

The intergroup comparison (Fig. 4b) only presented a statistical difference between the following groups: LE7 and LSE7 (p-value = 0.037), LE14 and LSE14 (p-value = 0.037) and L21 and LS21 (p-value = 0.007).

## Discussion

The present study analyzed the effect of FS, associated or not to AE, on the calcaneal tendon repair. Thus, our findings show that the isolated heterologous FS or AE decreased the EV, prevented tendon degenerative morphological modifications, and stimulated the tendon repair. This was observed by the decrease in the Bonar score, and in the increase of collagen – both positive contributions to the regenerative process. The association of FS with AE, during the acute phase of tendon repair, had the highest efficacy in reducing the EV, increasing the collagen ratio, decreasing the Bonar score, and accelerating the recovery process.

The incidence of tendon rupture had increased in the last four decades^2^ and it constitutes a big challenge to orthopedic medicine^4^. Literature reports that FS allows the use of heterologous fibrinogen in addition to the thrombin-like enzyme of snake venom. The thrombin-like enzyme transforms the fibrinogen molecule into fibrin monomers, which polymerizes in the presence of calcium to form a stable clot with adhesive, hemostatic, and sealant effects^33^.

The histopathological analysis of the present work showed that the FS did not induce tissue necrosis or the development of infections. Therefore, these results indicate that FS is a biocompatible material. Similarly, De Barros *et al.* 34analyzed cartilage repair, using the FS derived from rattlesnake venom, as scaffolding with excellent applicability. In his work, the FS gel did not trigger undesirable effects, such as inflammation, and allowed a normal repair process, confirming our results.

Additionally, the histopathological evaluation of the present study demonstrated tenocytes proliferation, granulation tissue, and collagen formation in the tendon partial transection area in the FS treated group. In the same way, Frauz *et al.* 4 investigated the use of FS, associated or not with mesenchymal stem cells, in the treatment of calcaneal tendon partial transection. The authors suggested the FS as a good option for treatment during tendon repair, due to its effectiveness in tendon organization recovery when compared to FS associated with mesenchymal stem cells on the 21^st^ day post injury.

Moreover, the literature showed that AE leads to an increase in the unnatural tissue strength if it starts immediately after the injury and during the inflammation peak. The present work started the AE 3 days after the partial transection of the calcaneal tendon. This therapy promoted EV reduction and stimulated the tendon when compared to control group. These findings are in agreement with the results presented by Sheikhani-Shahin *et al.* 35while it eventually causes various clinical problems. This study assessed the healing effect of bone marrow-derived stem cells (BMSCs. They analyzed the effect of AE on tendons lesions in rat and reported a significant increase in cellularity in the aquatic activity group, compared to the control group. Thus, it is possible to suggest that AE is capable of stimulating tendon repair by a mechanotransduction response.

In the present study, the FS and AE association demonstrated highest efficacy in reducing the EV, higher amount of granulation tissue, and increased collagen deposition at the site of the injury, contributing to accelerate the recovery process. Therefore, the use of FS associated to AE had the highest positive impact on the calcaneal tendon repair compared to the isolated use of FS or AE. Probably, the stimulus associations were able to stimulate tenocyte metabolism, subsequently affecting the increase of cell proliferation and the synthesis of structures that make up the tendon.

FS mimics the physiological blood clot formation and acts with a prompt reaction, involving fibrinogen to thrombin conversion. In addition to hemostasis, the fibrin clot and its cleavage products have regulatory effects on tissue healing during the injury-induced inflammatory processes.

According to Hsieh *et al*.^36^, fibrin is a fibrous protein resulting from blood clotting and it provides a temporary matrix in which cells migrates and adhere during wound healing. Macrophages are needed for both advancing and resolving inflammation process. The authors demonstrated that a culture of macrophages on fibrin matrices exerts an anti-inflammatory effect, whereas the soluble precursor fibrinogen stimulates inflammatory activation.

Moreover, culture on fibrin completely abrogates inflammatory signaling caused by fibrinogen, or inflammatory stimuli. Thus, the study of Hsieh *et al*.^36^ shows that the presentation of fibrin is important for regulating a switch between macrophage pro- and anti-inflammatory behavior. Furthermore, the aquatic environment is shown to be adequate for rehabilitation of calcaneal tendon injuries once it decreases gravitational load, allowing for early mobilization and exercises. During exercise, tendinous cells respond to mechanical stimulus (load) by producing growth factors (IGF-I, TGF-?1) and increasing the synthesis of tendinous collagen in animal experiments^23^. Collagen is the main constituent of tendons (60 to 90%) and is related to the structure and biomechanical properties of tendons recovery during the repair process^4^,^23^. A high expression of collagen is essential to obtain a faster healing of tendons^37^.

In view of the aforementioned, the association ofFS and AE therapies modulated the inflammatory process and increased the collagen deposition, culminating in the earlier resolution of the inflammatory process and earlier differentiation of tenocytes, accelerating the tendon healing process.

## Conclusions

Our findings suggest that both isolated FS and AE treatments were effective in preventing tendon degenerative morphological modifications. However, the association of FS and AE had the highest efficacy in accelerating the tendon recovery process. Finally, this was the first research using a heterologous fibrin biopolymer sealant associated with the aquatic exercise, opening a new possibility to apply in patients this new treatment since previously corroborated by clinical trials.
